# Mucoepidermoid carcinoma of the salivary glands revisited with special reference to histologic grading and *CRTC1/3-MAML2* genotyping

**DOI:** 10.1007/s00428-021-03146-x

**Published:** 2021-07-07

**Authors:** André Fehr, Sarah Werenicz, Pietro Trocchi, Markus Falk, Reinhard E. Friedrich, Angelika Stammler, Andreas Stang, Florian Oesterling, Laura Khil, Göran Stenman, Werner Böcker, Katharina Tiemann, Thomas Löning

**Affiliations:** 1 Sahlgrenska Center for Cancer Research, Department of Pathology, Sahlgrenska University Hospital, University of Gothenburg, Gothenburg, Sweden; 2 Department of Oral and Craniomaxillofacial Surgery, Eppendorf University Hospital, University of Hamburg, Hamburg, Germany; 3 Institute of Medical Informatics, Biometry and Epidemiology (IMIBE), University Hospital Essen, Essen, Germany; 4 Institute for Hematopathology, Hamburg, Germany; 5 Faculty of Medicine, Justus-Liebig-University Giessen, Giessen, Germany; 6 Cancer Registry North Rhine-Westphalia, Bochum, Germany; 7 Gerhard Seifert Reference-Center - Hansepathnet, Hamburg, Germany

**Keywords:** *CRTC1-MAML2*, Gene fusion, Head and neck neoplasms, Mucoepidermoid carcinoma; Neoplasm grading, Observer variation

## Abstract

Mucoepidermoid carcinoma (MEC) is the most common carcinoma of the salivary glands. Here, we have used two large patient cohorts with MECs comprising 551 tumors to study clinical, histological, and molecular predictors of survival. One cohort (n = 167), with known *CRCT1/3-MAML2* fusion status, was derived from the Hamburg Reference Centre (HRC; graded with the AFIP and Brandwein systems) and the other (n = 384) was derived from the population-based Cancer Registry of North Rhine-Westphalia (LKR-NRW; graded with the AFIP system). The reliability of both the AFIP and Brandwein grading systems was excellent (n = 155). The weighted kappa for inter-rater agreement was 0.81 (95% CI 0.65–0.97) and 0.83 (95% CI 0.71–0.96) for the AFIP and Brandwein systems, respectively. The 5-year relative survival was 79.7% (95% CI 73.2–86.2%). Although the Brandwein system resulted in a higher rate of G3-MECs, survival in G3-tumors (AFIP or Brandwein grading) was markedly worse than in G1/G2-tumors. Survival in > T2 tumors was markedly worse than in those with lower T-stage. Also, fusion-negative MECs had a worse 5-year progression-free survival. The frequency of fusion-positive MECs in the HRC cohort was 78.4%, of which the majority (86.7%) was G1/G2-tumors. In conclusion, the AFIP and Brandwein systems are useful in estimating prognosis and to guide therapy for G3-MECs. However, their significance regarding young age (≤ 30 years) and location-dependent heterogeneity of in particular G2-tumors is more questionable. We conclude that *CRTC1/3-MAML2* testing is a useful adjunct to histologic scoring of MECs and for pinpointing tumors with poor prognosis with higher precision, thus avoiding overtreatment.

**Supplementary Information:**

The online version contains supplementary material available at 10.1007/s00428-021-03146-x.

## Introduction

Mucoepidermoid carcinoma (MEC) is the most common salivary gland malignancy especially in young adults and children [1, 2]. The tumor is typically composed of mucinous, intermediate (clear cell), and squamoid cells forming cystic or solid patterns [2]. The predominant variant (i.e., cystic and differentiated) usually follows a favorable clinical course after surgical resection [3]. In contrast, the less common variant with a more solid architecture, necrosis, and prominent cellular/nuclear atypia is prone to invasive growth and metastases [4, 5]. Based on their histological appearances, pathologists at the Armed Forces Institute of Pathology (AFIP) established the first grading system for salivary MECs [6, 7]. However, this system and its later modifications continue to evoke critical reservations concerning the identification of true high-risk MECs [8].

Today, the two most frequently employed (semiquantitative) grading systems, the AFIP and Brandwein systems [9, 10], divide MEC into low- (G1), intermediate- (G2), and high-grade (G3) tumors assigning points to specific histologic features. Importantly, scoring of the same tumor is not always concordant between the two systems, especially regarding the distinction between G2- and G3-tumors. In contrast to AFIP, the Brandwein system weights different aspects of tumor invasion higher. Although the latter system is recommended by the latest WHO Classification of Head and Neck Tumors [2], there is still some debate about its clinical implications, in particular the risk of overscoring and thus overtreatment.

The role of molecular testing of salivary gland tumors was recently reviewed by Skalova and co-workers [11]. Previous studies have shown that the underlying molecular mechanism of MEC development is a recurrent t(11;19) translocation [12] resulting in a *CRTC1-MAML2* gene fusion [13, 14]. The fusion occurs at a very high frequency in MEC, mainly in G1- and G2-, and rarely in G3-tumors [15–17]. The discovery of this gene fusion and the rare variant fusion, *CRTC3-MAML2* [18], have refined the definition of this entity. Although early clinical studies claimed that patients with *CRTC1/3-MAML2*-negative MECs have a worse prognosis [15–17, 19, 20], this issue has not been finally resolved [2, 21].

The aim of this study was to investigate clinical, histological, and molecular predictors of survival of MEC patients in two large cohorts. We studied the association between age, sex, location, pT staging, grading, and the presence of the *CRTC1/3-MAML2* fusion. We also assessed the inter-rater variability of the AFIP and Brandwein grading systems and their impact on survival.

## Materials and methods

### Patient material

#### The Hamburg Salivary Gland Reference Centre cohort

The local Ethics Review Board approved the study in November 2017 (PV5412). Pathologists from the Hamburg Salivary Gland Reference Centre (HRC), in charged of the second opinion diagnoses of salivary gland tumors (TL and WB), routinely review all histological slides submitted by external laboratories. Whenever necessary, the HRC laboratory performs additional stainings (H&E, PAS, Alcian blue, and rarely immunohistochemical stainings). A series of 167 MECs of the major and minor salivary glands with adequate tumor material available, diagnosed between 2007 and 2020 was identified in the HRC archive. All cases were tested for the *CRTC1/3-MAML2* fusions as part of the routine diagnostic procedures. Clinical information for these cases were retrieved from the medical records at HRC or from the pathologist primarily in charge of the patient. Survival data was available for 60 patients. For 43 patients, the primary diagnosis dated back more than 5 years.

#### The population-based Cancer Registry of North Rhine-Westphalia cohort

The population-based cancer registry of North Rhine-Westphalia (LKR-NRW) in Germany represents the largest population-based cancer registry in Europe covering a population of 18.1 million inhabitants. All cancers of inhabitants of the Federal State of North Rhine-Westphalia are reported to LKR-NRW. Reporting of incident cancers occurs mainly through pathology reports, with an estimated completeness of cancer registration in 2016 of more than 90% [22]. We extracted all cases diagnosed between 2007 and 2017 with incident tumors morphologically coded as 8430/3 (MEC) and topographically coded as C00-C09 (malignant neoplasms of minor salivary glands, the parotid gland, and unspecified major salivary glands) according to the International Classification of Diseases for Oncology (ICD-O-3). The final population-based cohort included 384 MECs, for which all original reports were reviewed. Tumor grade was available in 332 cases (grading according to the AFIP system). Ninety-two of the 384 (24%) MECs were from the large Institutes of Pathology at the Universities of Münster, Bochum, Essen, Düsseldorf, Aachen, Köln, and Bonn. These centers have their own internal system for second opinions, and cases from these centers were therefore only randomly reviewed by the HRC experts. Cases from smaller institutions were reviewed by the HRC experts on demand. This was especially true for minor salivary gland MECs.

### Histologic reevaluation and fusion gene screening

We evaluated the inter-rater agreement by a blinded pathological reevaluation of 155 cases, exerted by two head and neck pathologists (TL and WB) according to the AFIP and Brandwein grading systems [9, 10]. The TNM-stage was defined based on the 8th edition of the AJCC guidelines [23]. All cases were screened for the *CRTC1-MAML2* fusion, by RT-PCR and sequenced as previously described [16]. In addition to *CRTC1-MAML2* fusion transcripts, our PCR assay also detects rare *CRTC3-MAML2* transcripts appearing as PCR prodcucts of atypical size. All of the latter PCR products were analyzed by direct Sanger sequencing [18].

### Statistical methods

To assess the inter-rater agreement between the pathologists grading according to both the AFIP and the Brandwein systems, we estimated observed and chance-corrected agreements (weighted kappa), including 95% confidence intervals, based on a dichotomization of the pathological assessments (low-/intermediate-grade, G1/G2 versus high-grade, G3). For comparison of prevalences, we calculated prevalence ratios with 95% confidence intervals.

For the HRC cohort, we estimated the 5-year cumulative progression-free survival (PFS) and the corresponding standard errors. Survival time was calculated as the time interval between the date of the first treatment and the date of disease progression. Patients without disease progression were right-censored at the date of death or the date of the last follow-up visit. For the LKR-NRW cohort, we calculated sex-stratified age-standardized incidence rates (age standard: old European Standard population) for the overall period 2007–2017 including the corresponding standard error (SE). The 5-year absolute and relative survival was estimated by tumor grade using the period approach [24]. We computed age-standardized relative survival estimates according to the approach of Brenner et al. [25]. Relative survival equals the ratio of the observed probability of survival to the survival in the general population, here the population of North Rhine-Westphalia, given the same age, sex, and calendar period (expected survival).

## Results

### Characteristics of the MEC cohorts

The HRC cohort comprised 167 patients, all of which had the primary tumor surgically resected. Only patients with G3-tumors underwent concurrent neck dissection, some of which also received adjuvant radiotherapy. Seven patients (4%) had regional neck metastases (N1 or N3) at the time of presentation, one of these G3-tumors was positive for the *CRTC1-MAML2* fusion. In addition, in seven cases (one G2- and six G3-MECs), tumor residues were discovered at the margins (R1). In 88 patients (53%), complete removal of the tumor was unequivocally confirmed (R0). In the remaining cases, all but one derived from minor salivary glands, doubts were raised (n = 72) with regard to the margins and they were accordingly reported to the clinicians as RX.

In the LKR-NRW cohort, all patients (n = 384) were surgically treated and the age-standardized incidence rate was 0.16 (SE = 0.01) per 100,000 person-years for both, men and women, during the period 2007–2017. The grading-specific sex ratios (m to f) of the age-standardized incidence rates were 0.73, 1.05, and 1.75 for G1-, G2-, and G3-MECs, respectively. In both cohorts, tumors graded as G1 were most frequent.

Based on the Brandwein grading system (reevaluated after initial AFIP grading), 127 of 167 patients in the HRC cohort had G1-, 16 had G2-, and 24 had G3-tumors. Overall, 158 (95%) of the tumors were classified as low-stage (pT1/pT2) tumors and nine as high-stage (> pT2) tumors. The low- versus high-stage tumors were distributed differently across the different tumor grades, pT1/pT2: 126 G1-, 13 G2-, and 20 G3-tumors versus > pT2: 1 G1-, 3 G2-, and 5 G3-tumors. Recurrences occurred in patients with G1- (n = 3) and G2-tumors (n = 2), all patients recovered from disease after repeated surgery. Four of these five recurrent G1/G2-MECs were positive for the *CRTC1-MAML2* fusion. Regardless of the grading system used, none of the G1- and G2-MECs developed distant metastases during follow-up and all these patients survived. In contrast, the three patients with G3-tumors (all fusion-negative, and G3 according to either the AFIP or Brandwein systems) progressed (one local and four lymph node events), and died with metastatic spread to internal organs.

In the HRC cohort, 20 of 167 (12%) patients were aged 30 years or younger, and eight were younger than 18 years at the time of diagnosis. Among the 20 cases, 19 were G1- and only one was a G3-MEC. All MECs were classified as pT1- or pT2-tumors except for one case that was pT4. All 20 cases were *CRTC1/3-MAML2* fusion-positive. In the follow-up cohort, all patients (n = 10) were free of disease and remained alive, including the two abovementioned patients with high-grade/high-stage MEC (Table 1). In the LKR-NRW cohort, 34 of 384 (9%) patients were aged 30 years or younger, including 12 younger than 18 years at the time of diagnosis. Twenty-five of these had G1-/G2- and two G3-tumors; for seven cases, no grade was available. Nineteen MECs were classified as pT1- or pT2-tumors and seven as > pT2; for the remaining eight cases, the stage was unknown. All patients were alive at follow-up except for one patient who died of pancreatic cancer.

**Table 1:** Clinicopathologic characteristics of the Hamburg Salivary Gland Reference Centre (HRC) and Cancer Registry of North Rhine Westfalia (LKR-NRW) cohorts

	HRC overall n = 167 (%)^a^	HRC follow-up n = 60 (%)^a^	LKR-NRW n = 384 (%)^a^
Time period	2007–2020	2007–2020	2007–2017
Age (years)			
Mean/median (range)	51.3/52 (4–90)	50.5/53.5 (4–84)	58.6/61 (4–94)
≤ 18	8 (5)	8 (13)	12 (3)
19–30	12 (7)	2 (3)	22 (6)
31–49	58 (35)	16 (27)	88 (23)
≥ 50	89 (53)	34 (57)	262 (68)
Sex			
Male	75 (45)	32 (53)	186 (48)
Female	92 (55)	28 (47)	198 (52)
Primary site			
Major gland	75 (45)	28 (47)	236 (61)
Minor gland	92 (55)	32 (53)	148 (39)
*CRTC1/3-MAML2*			
Positive	131^b^ (78)	47 (78)	-
Negative	36 (22)	13 (22)	-
Tumor grade			
G1	127 (76)	47 (78)	178 (46)
G2	16 (10)	5 (8)	73 (19)
G3	24 (14)	8 (13)	81 (21)
Not reported			52 (14)
pT-stage^c^			
T1	113 (68)	36 (60)	132 (34)
T2	45 (27)	21 (53)	83 (22)
T3	3 (2)	1 (2)	45 (12)
T4	6 (4)	2 (3)	30 (8)
TX	-	-	94 (24)
N-stage			
N0	51 (31)	27 (45)	159 (41)
N1	6 (4)	2 (3)	22 (6)
N3	1 (1)	1 (2)	44 (11)
NX	109 (65)	30 (50)	158 (41)
M-stage			
M0	48 (29)	25 (42)	120 (31)
M1	1 (1)	1 (2)	6 (2)
MX	118 (71)	34 (57)	258 (67)
R-status			
R0	88 (53)	35 (58)	-
R1	7 (4)	2 (3)	-
RX	72 (43)	23 (38)	-
Progression	9 (5)	9 (15)	-
Deaths			
Tumor-related	-	3 (5)	25 (7)
Unrelated death	-	5 (8)	92 (24)

^a^Percentages have been rounded and may not total 100

^b^Two cases with *CRTC3-MAML2* fusion

^c^8th AJCC Cancer Staging Manual

### Inter-rater agreement of the two grading systems

We evaluated the inter-rater agreement between two pathologists on the dichotomized grading (G1/G2 versus G3) of 155 cases. The prevalence of G3-tumors according to pathologist TL and pathologist WB were 9.7% and 9.0%, respectively. The overall observed and weighted kappa agreements between the pathologists based on the AFIP system were 0.97 (95% CI: 0.93–0.99) and 0.81 (95% CI: 0.65–0.97), respectively. The corresponding agreements based on the Brandwein system were 0.96 (95% CI: 0.92–0.99) and 0.83 (95% CI: 0.71–0.96), respectively. The kappa agreements were lower for the AFIP than for the Brandwein system among cases derived from major salivary glands (0.68, 95% CI: 0.39–0.97 versus 0.89, 95% CI: 0.74–1.0) and among patients aged 0–49 years (0.66, 95% CI: 0.04–0.96 versus 0.79, 95% CI: 0.40–1.0). Among the minor salivary gland MECs, the kappa agreement was higher for the AFIP than for the Brandwein system (0.93, 95% CI: 0.79–1.0 versus 0.77, 95% CI: 0.56–0.99) (Supplementary Table).

### Concordance of the AFIP and Brandwein grading systems

The concordance of grading based on the AFIP and the Brandwein systems was 94.8% and 91.6% for pathologist TL and WB, respectively. The Brandwein grading tended to produce a higher percentage of G3-tumors compared to the AFIP system (Table 2).

**Table 2:** Grading of 155 MECs according to the AFIP and Brandwein systems by two blinded pathologists

Pathologist TL	Brandwein						Overall	%
	G1		G2		G3			
	N	%	N	%	N	%	N	
AFIP								
G1	119	76.8	2	1.3	0	0.0	121	78.1
G2	0	0.0	13	8.4	6	3.9	19	12.3
G3	0	0.0	0	0.0	15	9.7	15	9.7
Overall	119	76.8	15	9.7	21	13.5	155	
Concordance (%)				94.8				
Pathologist WB								
G1	120	77.4	6	3.9	3	1.9	129	83.2
G2	0	0.0	8	5.2	4	2.6	12	7.7
G3	0	0.0	0	0.0	14	9.0	14	9.0
Overall	120	77.4	14	9.0	21	13.6	155	
Concordance (%)				91.6				

Percentages are cell percentages with denominator n = 155 MECs

### CRTC1/3-MAML2 fusion status

In total, 131 of 167 cases (78.4%) from the HRC cohort were positive for *CRTC1/3-MAML2* fusions (Table 3). Fusions were found in 115 G1-, nine G2-, and seven G3-tumors. Ninety-four of the fusion-positive patients had pT1-, 35 pT2-, one pT3-, and one pT4-disease. Of the 131 fusion-positive patients, 78 were females and 53 were males.

**Table 3:** Prevalence of the *CRTC1/3-MAML2* fusion by grade and stage in the Hamburg Salivary Gland Reference Centre (HRC) overall (n = 167) and follow-up cohorts (n = 60)

	*CRTC1/3-MAML2* fusion		Prevalence ratio of fusion presence and 95% CI*	
	Fusion-negative (%)	Fusion-positive (%)	Ratio	95% CI
HRC overall	36 (21.6)	131 (78.4)		
Tumor grade				
G1–G2	19	124 (86.7)	3.0	1.6–5.6
G3	17	7 (29.2)	Ref	
pT-stage				
T1	19	94 (83.2)	1.2	1.0–1.5
> T1	17	37 (68.5)	Ref	
HRC follow-up	13 (21.7)	47 (78.3)		
Tumor grade				
G1–G2	7	45 (86.5)	3.5	1.0–11.6
G3	6	2 (25.0)	Ref	
pT-stage				
T1	5	31 (86.1)	1.3	1.0–1.8
> T1	8	16 (66.7)	Ref	

^*^CI, confidence interval

**Fig. 1:**
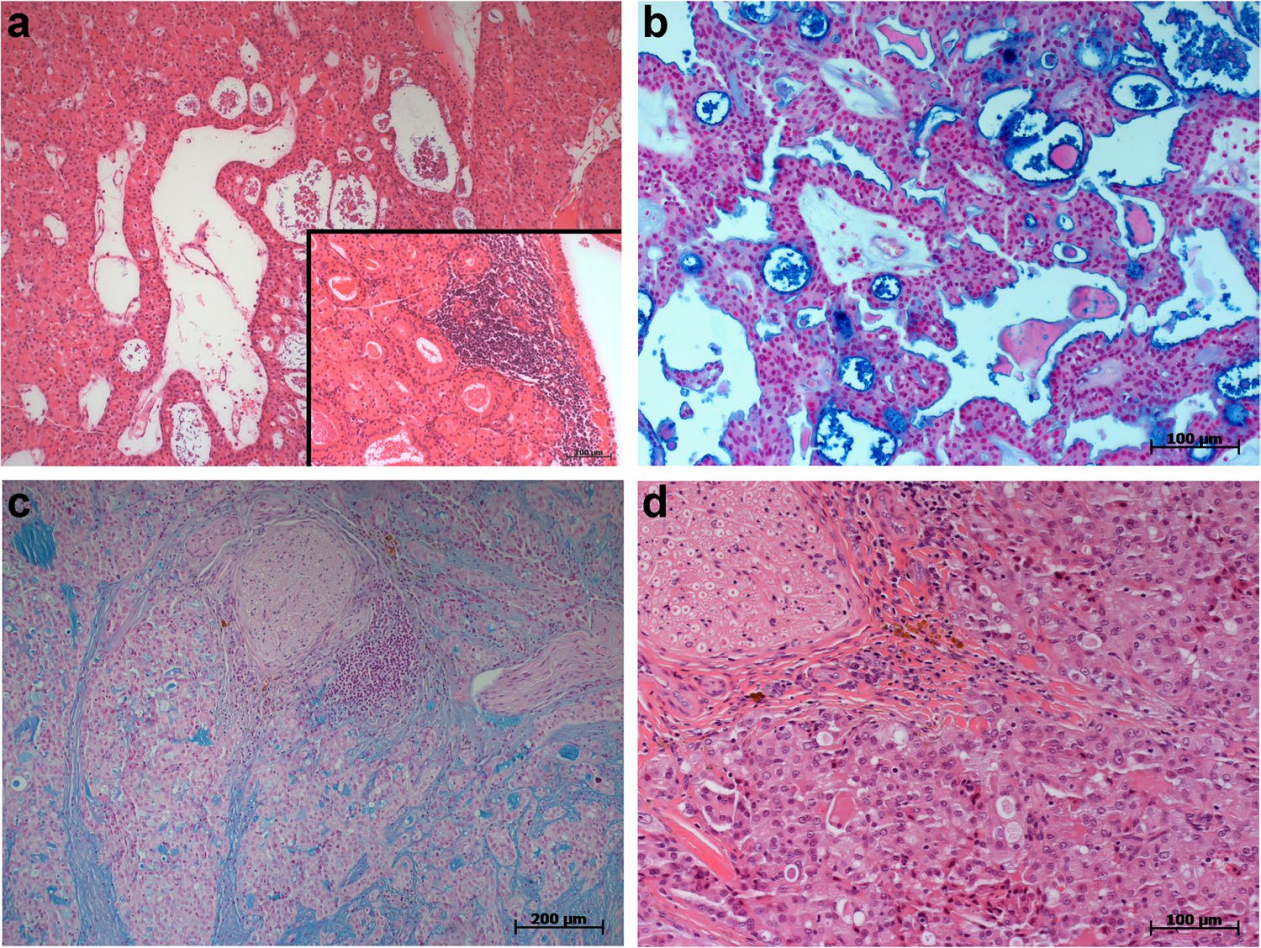
H&E- and Alcian blue–stained MECs of the parotid gland. **a** Oncocytic variant with mixed solid and cystic architecture und typical hyper-eosinophilic phenotype, grade 1 (*CRTC1-MAML2* positive; H&E × 100). Inset: recurrent Warthin-like MEC of the palatal mucosa (*CRTC1-MAML2* positive; H&E × 200). **b** Same oncocytic variant as in **a** (Alcian blue × 200). **c** Parotid gland MEC with mainly solid architecture and typical mixture of intermediate and mucus-producing cells (goblet cells). Clearly invasive phenotype with perineural growth, grade 3 (*CRTC1-MAML2* negative, Alcian blue × 100). **d** Same as in **c** showing different degrees of nuclear atypia (H&E × 200)

In the HRC follow-up cohort, 47 of the 60 MECs (78.3%) were fusion-positive. G1–G2-tumors had a 3.0-fold prevalence of the fusion compared to G3-tumors (prevalence ratio 3.0, 95% CI: 1.6–5.6). The fusion was slightly more prevalent among tumors staged pT1 compared to higher stages (prevalence ratio 1.2, 95% CI: 1.0–1.5). All young patients (≤ 30 y, n = 20) were fusion-positive. Three oncocytic MECs were G1 and fusion-positive (Fig. 1a and b), one clear cell MEC was G1 and fusion-negative, and one recurrent Warthin-like MEC was G2 and fusion-positive (Fig. 1a). Of the two noninvasive G3-MEC-ex-pleomorphic adenomas (MEC-ex-PAs), one was fusion-positive (Fig. 1c and d). Only two MECs (both G1) were positive for the *CRTC3-MAML2* fusion variant*.*

### Survival

**Fig. 2:**
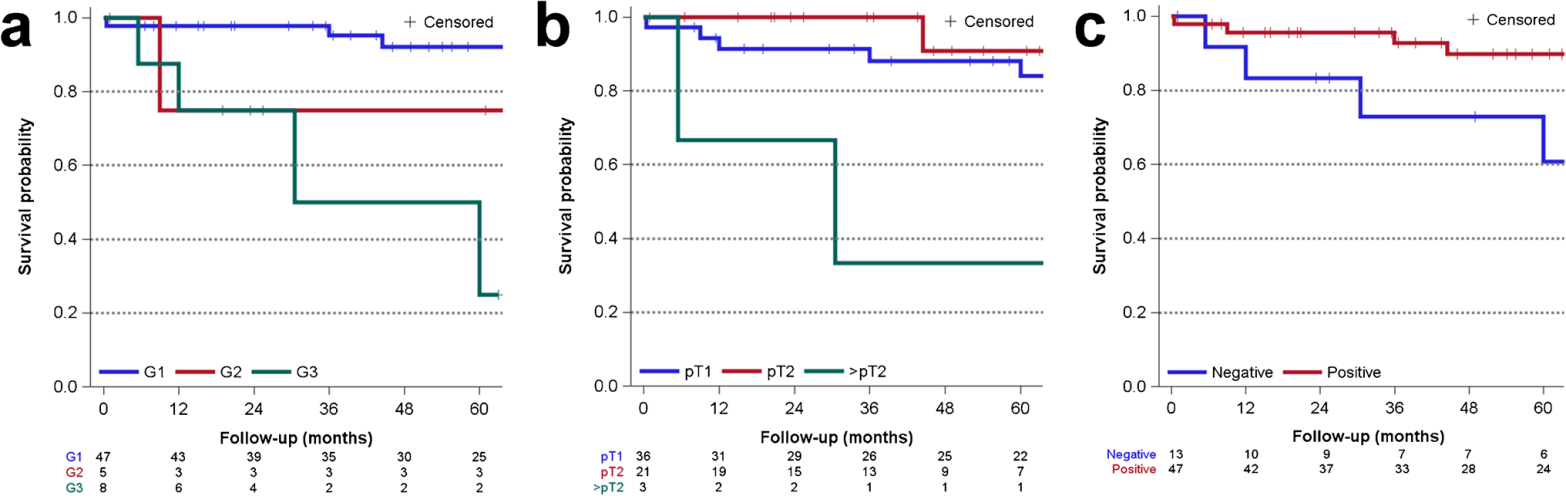
Kaplan–Meier curves for the Hamburg Salivary Gland Reference Centre follow-up (n = 60) cohort. Progression-free survival according to **a** grade, **b** T-stage, and **c** *CRTC1/3-MAML2* status

The estimated 5-year cumulative probability of progression-free survival (PFS) in the HRC follow-up cohort (n = 60) was 86% (SE = 0.05). The probability was considerably lower for high-grade tumors (50%, SE = 0.23) than for low-grade tumors (91%, SE = 0.04). The PFS probabilities for G1-, G2-, and G3-graded cases were 92% (SE = 0.04), 75% (SE = 0.21), and 50% (SE = 0.23), respectively. Stage-specific PFS was 88% (SE = 6%) and 91% (SE = 9%) for pT1- and pT2-tumors, respectively. Although based on small numbers, PFS was considerably lower for pT3-tumors (33%, SE = 27%). PFS was higher among patients with the *CRTC1/3-MAML2* fusion (90%, SE = 5%) compared to fusion-negative patients (73%, SE = 14%) (Fig. 2).

**Fig. 3:**
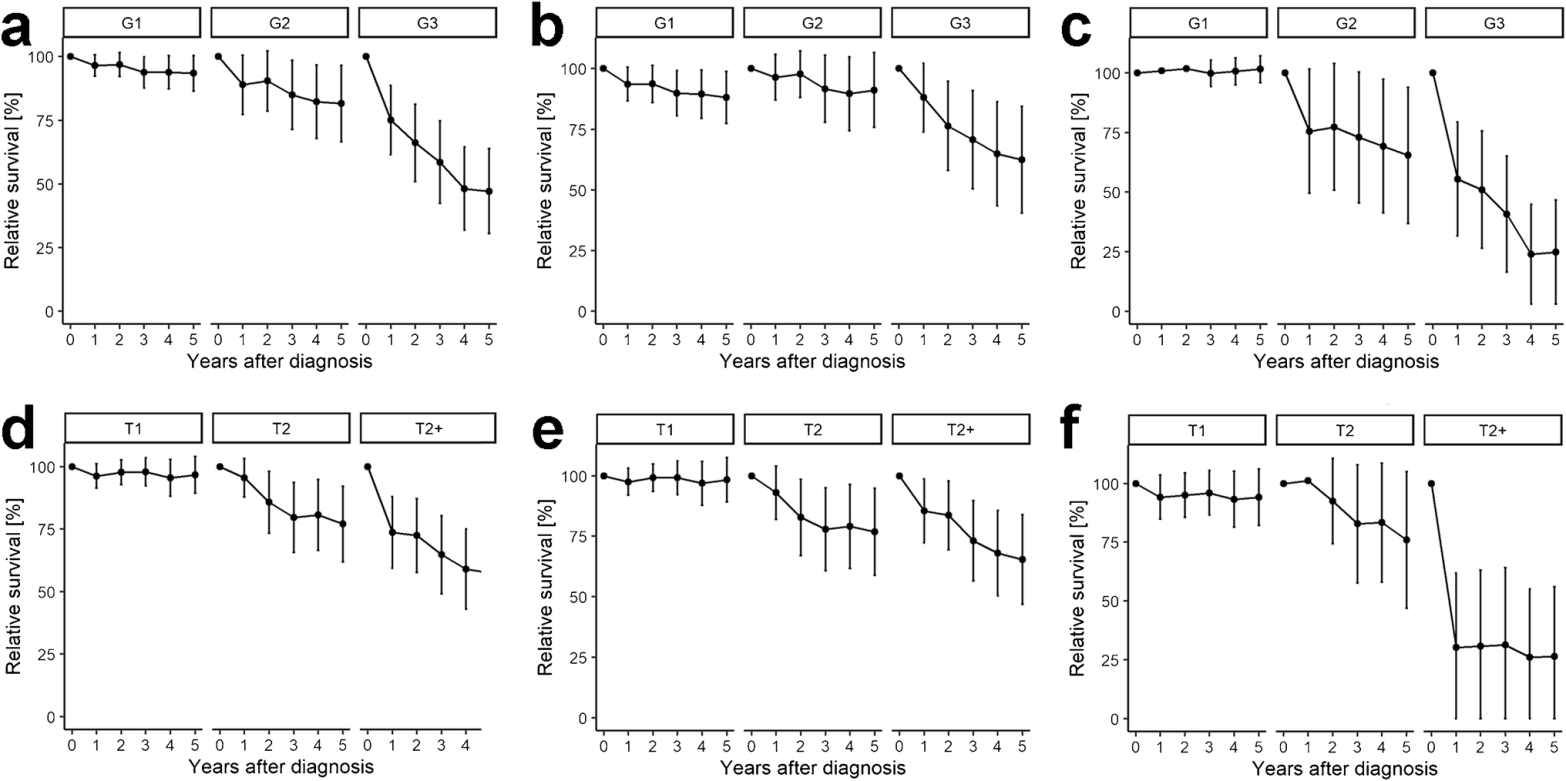
Grading- and T-stage-specific relative survival of patients with salivary gland MECs in the LKR-NRW cohort (2007–2017). **a** and **d** Complete cohort. **b** and** e** Major salivary glands (ICD-10 C07 and C08). **c** and **f** Minor salivary glands (ICD-10 C00-C09 without C07 and C08)

Among the 332 cases from the LKR-NRW cohort with grading and follow-up available, the absolute 5-year survival for G1- (n = 178), G2- (n = 73), and G3- (n = 81) tumors were 87.7% (SE = 3.3), 77.4% (SE = 7.0), and 40.2% (SE = 7.3), respectively. The corresponding relative survival estimates were 93.5% (SE = 3.6), 81.6% (SE = 7.7), and 47.1% (SE = 8.5), respectively. The survival trends for G1- and G2-MEC patients were very similar, whereas the survival for G3-MEC patients were considerably lower during the 5 years of follow-up (Fig. 3a). The survival curves of patients with MEC of major salivary glands closely resembled those of the complete cohort with a markedly worse prognosis for G3-tumors compared to G1-tumors (Fig. 3b). Among minor salivary gland MECs, patients with G1-tumors had a relative survival of about 100%, whereas the relative survival of patients with G2- and G3-tumors were much worse (Fig. 3c).

With regard to pT-stage, the survival analysis showed a similar picture. The survival trend for pT1-staged MECs within the complete cohort was excellent and for pT2-tumors good, whereas the survival for pT3/pT4-staged MECs were considerably lower during the last 3 years of follow-up (Fig. 3d). The survival curves of patients with major salivary gland MECs closely resembled those of the complete cohort (Fig. 3e). In patients with minor salivary gland MEC, the relative survival of pT3/pT4-staged tumors was worse during the 5 years of follow-up (Fig. 3f).

## Discussion

In this study, we have investigated clinical, histological, and molecular predictors of survival of MEC patients in two large patient cohorts, one from the HRC pathology (n = 167) and one from the population-based LKR-NRW cancer registry (n = 384). We present up-to-date survival estimates of MECs of the major and minor salivary glands. Survival is clearly worse among cases with a higher stage (> T2) and grade (G3), and absence of the *CRTC1/3-MAML2* fusion gene. Notably, MECs of young patients (≤ 30 years) follow an entirely different clinical course. All 10 young patients in the HRC follow-up subgroup (all fusion-positive) and all 33 patients of the same age group in the LKR-NRW cohort had a favorable outcome independent of stage or grade. These observations are in line with previous studies showing that the *CRTC1/3-MAML2* fusion occurs with a frequency of up to 100% in young patients and is associated with a favorable outcome, also in patients with high-stage (> pT2) and high-grade (G3) disease [1, 26].

Interestingly, as previously shown by Taylor et al., men with MECs of the major salivary glands have shorter survival than women which may be due to a higher proportion of G3-MECs among men compared to women [27]. In contrast to Taylor et al., we also studied the survival of patients with minor salivary gland MECs. In the LKR-NRW cohort, survival steadily decreased from G1 to G3 in minor salivary gland tumors. The G1-MECs showed a relative survival of nearly 100%, whereas the survival was significantly shorter for G2- and G3-tumors. This observation partly contradicts the study by Navale et al. which shows that minor salivary gland MECs have an increased tendency to metastasize even in the presence of histologic and molecular genetic features that would predict an indolent behavior [28]. It should, however, be noted that the number of cases analyzed is limited and that we lack data on the relative survival rate of different grades. Therefore, the survival rate of G1 patients might be higher than expected.

Overall, the inter-rater agreements between the AFIP and Brandwein grading systems were excellent. As expected, the Brandwein grading resulted in a higher percentage of G3-tumors, suggesting a possible increased risk of overtreatment [29]. Although this argument cannot be entirely excluded, our data show no grading-specific survival advantages for patients in the HRC cohort (re-graded according to the Brandwein system) compared with the LKR-NRW cohort (graded according to the AFIP system). Importantly, the presence of the *CRTC1/3-MAML2* fusion gene was strongly associated with grading; G1/G2-tumors were more frequently fusion-positive compared to G3-tumors.

Our results corroborate the notion that the Brandwein grading system considers the invasive phenotype as an additional and important grading criterion of MEC [30]. In our view, however, the inherent failures of this and other three-tiered grading systems are not resolved by using a binary modification (G1/G2 versus G3) as recommended by Cipriani et al. [31], because it does not eliminate the dilemma of a grey zone within the G1/G2-subgroups. This dilemma gets even more pronounced when looking separately at the survival of MECs of the major and minor salivary glands. The survival of patients with G2-MECs of the major glands was very similar to that of patients with G1-tumors. For patients with minor gland MECs, the survival declined steadily from G1- to G3-tumors. One explanation for this observation is that tumors of the minor glands are diagnostically more challenging, since they are almost invariably removed in multiple fragments to preserve anatomical and functional structures. This predicament not only compromises the quality of the histological diagnosis, but may also lead to an increase in the proportion of MECs graded as G2/G3. It also elicits doubts about clear margins of the resection specimens, which explains the high number of RX-cases in the HRC cohort.

Only two MECs were initially misinterpreted in the HRC cohort, one recurrent Warthin-like MEC of the palate (G2 and fusion-positive) and one adenocarcinoma with a minor mucinous component (G3 and fusion-negative). The latter was subsequently excluded after reevaluation because of its exclusive luminal phenotype. These observations are in line with previous studies and establish the value of *CRTC1/3-MAML2* fusion testing for diagnostic purposes [16, 17, 32]. This is not only true for the identification of bona fide carcinomas, but also for the differential diagnosis of benign tumors such as oncocytomas and Warthin tumors (MEC-like variants) and for certain cystic lesions (congenital or acquired) with mucous metaplasia [13, 33–40]. In the HRC cohort, we noticed three oncocytic MEC variants, one clear cell variant, one Warthin-like variant, and two noninvasive MEC-ex-PA. Among these seven cases, five were positive for *CRTC1-MAML2*, including the abovementioned recurrent Warthin-like MEC of the palate. Our data clearly demonstrates that most G1- and G2-MECs are fusion-positive, whereas G3-MECs are mostly negative [16, 17]. We found only two MECs with the *CRTC3-MAML2* fusion variant, which confirms the rarity of this fusion [18, 19]. Collectively, our findings further emphasize previous observations from smaller series of MECs that the presence of the *CRTC1/3-MAML2* fusion is associated with favorable clinical features, low-grade tumor histology, and a good prognosis [15–17, 19, 20].

It should be emphasized that the differential diagnosis of G3-MECs is difficult even for experienced head and neck pathologists. Misinterpretations may occur when the number of mucous-producing cells is low or when these cells are hidden in structures of more or less undifferentiated squamous cells, leading to the diagnosis of, in particular, squamous cell carcinoma. Other differential diagnoses to consider are, for example, salivary duct carcinomas [17, 20] and adenosquamous carcinomas (the latter is not included in the latest WHO Classification of Head and Neck Tumors [2]). Interestingly, two recent studies of adenosquamous carcinomas of the pancreas showed that a considerable number of these cases (43.2% and 36%), in addition to the classical pancreatic ductal adenocarcinoma component, also contained a high-grade MEC component [41, 42]. *MAML2* fusion gene testing in one of these cohorts revealed that all tumors were fusion-negative [42]. These types of mixed carcinomas were never seen in the present G3 salivary MECs.

We are fully aware that the combination of a population-based (LKR-NRW) and a consultation-based series (HRC) is unusual and carries potential biases. However, both cohorts are coherent since all pathologists used the same diagnostic principles and AFIP grading rules (trained in courses by the German Section of the IAP over decades). Thus, we conclude that the two cohorts are much more homogeneous than initially expected, with the possible exception of G2-MECs of minor glands which more often were classified as G3-tumors in the LKR-NRW-series. Finally, re-grading of the MECs in the HRC cohort using the Brandwein system (initially graded using the AFIP system) resulted in no major changes in progression-free survival.

In summary, our findings demonstrate that both the AFIP and Brandwein grading systems reflect the clinical behavior of G1- and G3-MECs quite well. For the challenging G2-tumors, we recommend that molecular testing for the *CRTC1/3-MAML2* fusion is performed. Our findings show that detection of the fusion provides useful information for diagnosis, albeit it is not a powerful predictor of outcome. We conclude that *CRTC1/3-MAML2* testing is a useful adjunct to histologic scoring of MECs and for pinpointing tumors with poor prognosis with higher precision, thus avoiding overtreatment. Continued clinical and molecular studies of large and well-characterized patient cohorts may eventually lead to the development of new clinical guidelines for the management of MEC patients.

## Supplementary Information

Below is the link to the electronic supplementary material.

Supplementary file1 (PDF 25 KB)

## Data Availability

HRC: The use of clinical data and archived diagnostic leftover tissues for research purposes has been approved by local laws (HmbKHG, §12,1) and by the local ethics committee (Ethics Commission Hamburg, PV5412). All work has been carried out in compliance with the Helsinki Declaration. Because of data protection regulations in Germany, data of the HRC can only be made available upon request with justification. NRW: In accordance with the State Cancer Registry Act of the State of North Rhine-Westphalia, data from the registry can only be made available upon request with justification.

## Abbreviations

MEC: Mucoepidermoid carcinoma(s)
HRC: Hamburg Salivary Gland Reference Centre, Germany
LKR-NRW: Cancer Registry of North Rhine-Westphalia, Germany
PFS: Progression-free survival
SE: Standard error
