# Supplementary material for: Mucoepidermoid carcinoma of the salivary glands revisited with special reference to histologic grading and CRTC1/3-MAML2 genotyping
Source: Virchows Arch. 2021 Jul 7;479(5):975–85. doi: 10.1007/s00428-021-03146-x (PMC8572836; doi:10.1007/s00428-021-03146-x)
Supplement: Supplementary file 1 — Supplementary file1 (PDF 25 KB) [file 428_2021_3146_MOESM1_ESM.pdf]

Supplementary Information

Virchows Archiv

Mucoepidermoid carcinoma of the salivary glands revisited with special reference to histologic grading and *CRTC1/3-MAML2* genotyping

André Fehr[1#*], Sarah Werenicz[2#], Pietro Trocchi[3#], Markus Falk[4], Reinhard E. Friedrich[2], Angelika Stammler[5], Andreas Stang[3,6], Florian Oesterling[6], Laura Khil[6], Göran Stenman[1], Werner Böcker[7], Katharina Tiemann[4], Thomas Löning[7]

equal authorship

[1] Sahlgrenska Center for Cancer Research, Department of Pathology, University of Gothenburg, Sahlgrenska University Hospital, Gothenburg, Sweden

[2] Department of Oral and Craniomaxillofacial Surgery, Eppendorf University Hospital, University of Hamburg, Hamburg, Germany

[3] Institute of Medical Informatics, Biometry and Epidemiology (IMIBE), University Hospital Essen, Essen, Germany

[4] Institute for Hematopathology, Hamburg, Germany

[5] Faculty of Medicine, Justus-Liebig-University Giessen, Giessen, Germany

[6] Cancer Registry North Rhine-Westphalia, Bochum, Germany

[7] Gerhard Seifert Reference-Center - Hansepathnet, Hamburg, Germany

*** Corresponding author**

André Fehr: andre.fehr@gu.se

Supplementary Table Interobserver agreement of the grading (high- vs. low-) of 155 mucoepidermoid carcinomas between two pathologists according to potential modifiers of the agreement

	**AFIP Agreement (95%CI*)**		**Brandwein Agreement (95%CI)**	
	**Observed**	**Weighted Kappa**	**Observed**	**Weighted Kappa**
**Overall** (n=155)	0.97 (0.93-0.99)	0.81 (0.65-0.97)	0.96 (0.92-0.99)	0.83 (0.71-0.96)
**Age at diagnosis (years)**				
0-49 (n=77)	0.99 (0.93-1.0)	0.66 (0.04-1.0)	0.99 (0.93-1.0)	0.79 (0.40-1.0)
50+ (n=78)	0.95 (0.87-0.99)	0.82 (0.66-0.99)	0.94 (0.86-0.98)	0.82 (0.67-0.97)
**Stage**				
pT1 (n=108)	0.98 (0.93-1.0)	0.74 (0.39-1.0)	0.97 (0.92-0.99)	0.81 (0.60-1.0)
>pT1 (n=47)	0.94 (0.82-0.99)	0.82 (0.61-1.0)	0.94 (0.82-0.99)	0.84 (0.66-1.0)
**Anatomic Site**				
Major glands (n=69)	0.94 (0.86-0.98)	0.68 (0.39-0.97)	0.97 (0.90-1.0)	0.89 (0.74-1.0)
Minor glands (n=86)	0.99 (0.94-1.0)	0.93 (0.79-1.0)	0.95 (0.89-0.99)	0.77 (0.56-0.99)
**Gene Fusion**				
Positive (n=124)	0.99 (0.96-1.0)	0.88 (0.66-1.0)	0.98 (0.96-1.0)	0.83 (0.59-1.0)
Negative (n=31)	0.87 (0.70-0.96)	0.71 (0.44-0.97)	0.87 (0.70-0.96)	0.74 (0.51-0.98)

* CI, confidence interval
